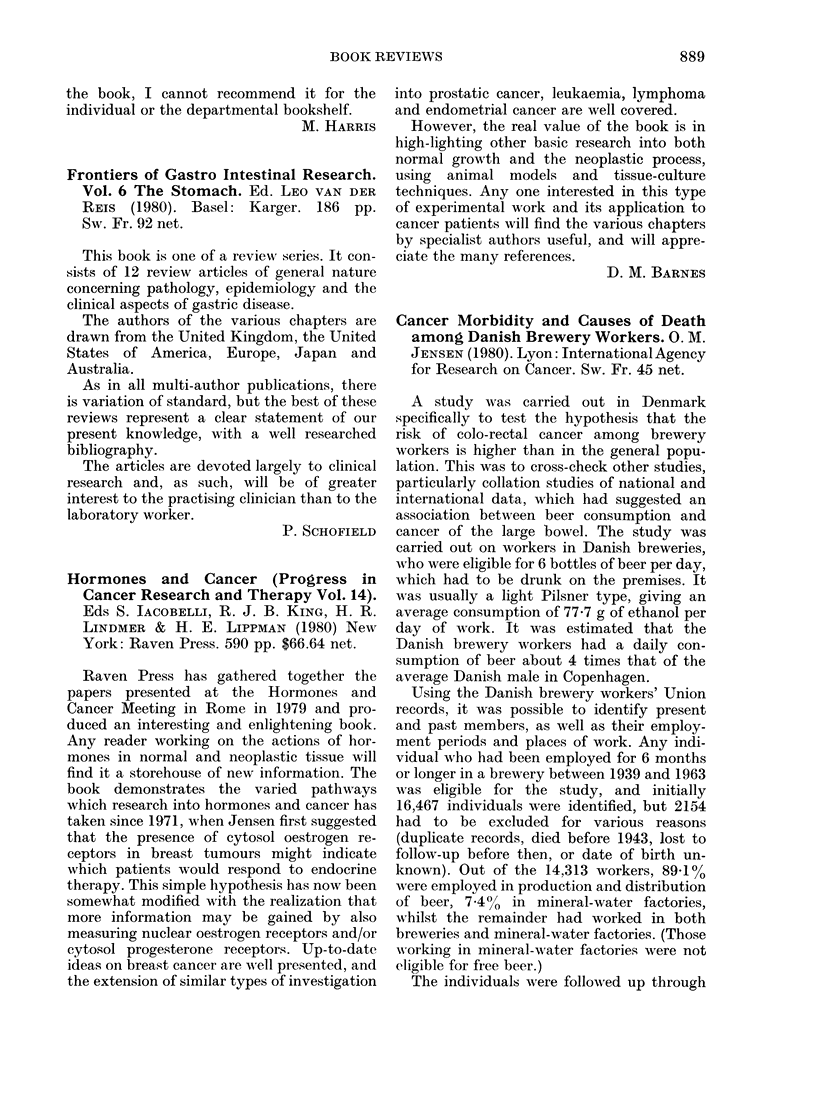# Hormones and Cancer (Progress in Cancer Research and Therapy Vol. 14)

**Published:** 1981-06

**Authors:** D. M. Barnes


**Hormones and Cancer (Progress in Cancer Research and Therapy Vol. 14).** Eds S. IACOBELLI, R. J. B. KING, H. R. LINDMER & H. E. LIPPMAN (1980) New York: Raven Press. 590 pp. $66.64 net.

Raven Press has gathered together the papers presented at the Hormones and Cancer Meeting in Rome in 1979 and produced an interesting and enlightening book. Any reader working on the actions of hormones in normal and neoplastic tissue will find it a storehouse of new information. The book demonstrates the varied pathways which research into hormones and cancer has taken since 1971, when Jensen first suggested that the presence of cytosol oestrogen receptors in breast tumours might indicate which patients would respond to endocrine therapy. This simple hypothesis has now been somewhat modified with the realization that more information may be gained by also measuring nuclear oestrogen receptors and/or cytosol progesterone receptors. Up-to-date ideas on breast cancer are well presented, and the extension of similar types of investigation into prostatic cancer, leukaemia, lymphoma and endometrial cancer are well covered.

However, the real value of the book is in high-lighting other basic research into both normal growth and the neoplastic process, using animal models and tissue-culture techniques. Any one interested in this type of experimental work and its application to cancer patients will find the various chapters by specialist authors useful, and will appreciate the many references.

D. M. BARNES